# PTC located in the upper pole is more prone to lateral lymph node metastasis and skip metastasis

**DOI:** 10.1186/s12957-020-01965-x

**Published:** 2020-07-28

**Authors:** Yi Dou, Daixing Hu, Yingji Chen, Wei Xiong, Qi Xiao, Xinliang Su

**Affiliations:** Department of Endocrine and Breast Surgery, the First Affiliated Hospital of Chongqing Medical University, Chongqing, 400016 China

**Keywords:** Papillary thyroid carcinoma (PTC), Lymphatic metastasis, Tumour location, Lymph node dissection

## Abstract

**Background:**

Lateral lymph node metastasis (LLNM) is very common in papillary thyroid carcinoma (PTC). The influence of tumour location on LLNM remains controversial. The purpose of this study was to reveal the association between PTC tumours located in the upper pole and LLNM.

**Methods:**

We reviewed a total of 1773 PTC patients who underwent total thyroidectomy with central and lateral lymph node dissection between 2013 and 2018. Patients were divided into two groups according to tumour location. Univariate and multivariate analyses were performed to identify risk factors associated with LLNM and “skip metastasis”.

**Results:**

In the upper pole group, LLNM and skip metastasis were significantly likely to occur. Multivariate analysis showed that tumours located in the upper pole, male sex, extrathyroidal extension (ETE), central lymph node metastasis (CLNM) and tumour size were independent risk factors for LLNM, with odds ratios ([ORs], 95% confidence intervals [CIs]) of 2.136 (1.707–2.672), 1.486 (1.184–1.867), 1.332 (1.031–1.72), 4.172 (3.279–5.308) and 2.496 (1.844–3.380), respectively. Skip metastasis was significantly associated with the primary tumour location in the upper pole and age > 55 years, with ORs of 4.295 (2.885–6.395) and 2.354 (1.522–3.640), respectively.

**Conclusions:**

In our opinion, papillary thyroid tumours located in the upper pole may have an exclusive drainage pathway to the lateral lymph nodes. When the tumour is located in the upper pole, lateral neck dissection should be evaluated meticulously.

## Introduction

The morbidity of thyroid carcinoma is increasing worldwide. From 2011 to 2015, 63,324 patients were diagnosed with thyroid carcinoma in America [1]. In Europe, the incidence of thyroid cancer in women was three times higher than that in men in 2012 according to data from the Cancer Registry [2]. In China, the incidence rates of thyroid carcinoma ranked third for females in 2015 [3]. Papillary thyroid carcinoma (PTC) has an excellent prognosis, with 10-year survival rates ranging from 90 to 97% [4, 5]. In PTC, however, lymph node metastasis is common in the early stage and is related to an increased risk of local-regional recurrence and the reoperation rate [6, 7]. Many previous studies have reported that a tumour located in the upper pole was an independent risk factor for lymph node metastasis [8, 9]. YK et al. [10] found that upper pole tumours had a pooled odds ratio (OR) of 2.96 (95% confidence interval [CI] = 1.93–4.53, *P* < 0.001) and were significantly associated with lateral lymph node metastasis (LLNM). Moreover, skip metastasis (LLNM without central lymph node metastasis [CLNM]) was also significantly associated with the primary tumour location in the upper pole, as reported by several study [11]. A report by Lei et al. [12] showed that primary tumours in the upper pole had an OR of 18.495 and were associated with skip metastasis in 450 PTC patients. These studies indicate that the regular pattern of lymph node metastasis in PTC is difficult to predict due to the different tumour locations. In the present study, we aimed to reveal the association between PTC located in the upper pole and LLNM and propose an independent lymph node drainage pathway.

## Materials and methods

### Patients

A retrospective analysis of consecutive patients with PTC who underwent surgery at the Department of Endocrine and Breast Surgery of the First Affiliated Hospital of Chongqing Medical University between January 2013 and December 2018 was performed. The study was approved by the Medical Ethics Committee of The First Affiliated Hospital of Chongqing Medical University (2020-218). Consent has been obtained from each patient or subject after full explanation of the purpose and nature of all procedures used. Patients who met the following criteria were excluded: incomplete clinicopathologic data, other types of thyroid carcinoma, underwent a reoperation, tumour occupying the whole lobe, a bilateral tumour and an isthmus tumour and distant metastasis. Based on these criteria, 1773 patients with PTC were included.

All patients were diagnosed through fine-needle aspiration biopsy (FNAB) before surgery, and the diagnosis was confirmed by pathological biopsy as PTC. Physical examinations, ultrasonography and fibrolaryngoscopy were routinely performed on each patient. All patients underwent total thyroidectomy (TT) with central compartment lymph node dissection (LND) and ipsilateral therapeutic or prophylactic lateral neck LND, of which most were therapeutic dissection due to clinical lymph nodes positive(cN1). Prophylactic dissection was performed in few patients with risk factors for lateral lymph node metastasis (extrathyroidal extension, central lymph node metastasis, intraoperative findings and so on). The central lymph nodes and lateral lymph nodes (including levels II, III and IV) were divided into different regions and examined pathologically by three pathologists. Tumour location was categorized as upper pole, middle pole, and lower pole based on the preoperative ultrasound report from two experienced ultrasound doctors and the findings obtained during the operation. Since there is no clear anatomical division or guideline, the thyroid glands are bisected into three equal volumes (upper pole, middle pole and lower pole) according to the consensus of most medical center. Tumours occupying more than one single pole were included in the upper pole group when the upper pole was involved.

### Clinicopathological variables

Tumour multifocality, Hashimoto’s thyroiditis (HT) and extrathyroidal extension (ETE) were confirmed by a pathological examination. The *t* test, the chi-squared test or Fisher’s exact test was used to compare differences in demographic and pathologic data on sex (male, female), age (≤ 55 years, > 55 years), tumour size (≤ 10 mm, between 10 and 20 mm and >20 mm), tumour location (upper/middle/lower pole), HT (yes, no), multifocality (yes, no) and ETE (yes, no) between the patients with or without LLNM. Logistic regression was used for multivariate analysis to estimate the statistical significance. All variables with significant differences in the univariate analysis were included in the multivariate analysis. Statistical significance was defined as *P* < 0.05, and the analyses were performed using SPSS version 25.0 (SPSS Inc., Chicago, IL, United States) and GraphPad 8.0.

## Results

### Clinicopathologic characteristics

**Fig. 1:**
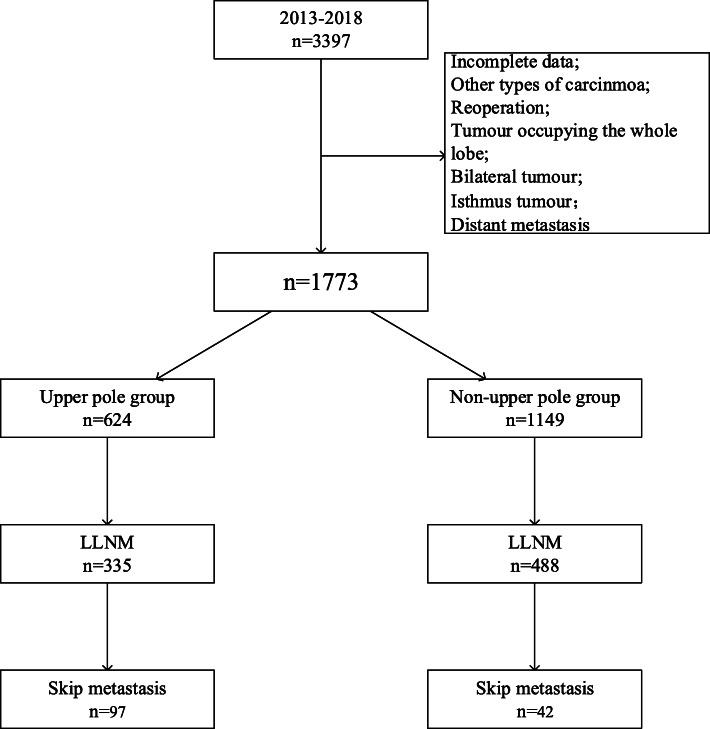
Flow diagram of all patients who underwent lateral neck dissection. LLNM, lateral lymph node metastasis; skip metastasis, lateral lymph node metastasis without central lymph node metastasis

Of the 1773 PTC patients included, 513 (28.9%) were male, and 1260 (71.1%) were female. The male-to-female ratio was 1:2.46. The median age of the patients was 42.1 ± 12.4 years. The mean size of the largest primary thyroid tumour was 1.5 ± 1.0 cm. Primary tumours located in the upper pole were found in 624 (35.2%) patients. A total of 862 (48.6%) patients had a tumour smaller than 1 cm, 624 (35.2%) had a tumour between 1 and 2 cm and 287 (16.2%) had a tumour larger than 2 cm. Gross ETE was observed in 375 (21.2%) patients. HT was present in 247 (13.9%) patients, and multifocal tumours were present in 312 (17.6%) patients. The analysis indicated that 1197 (67.5%) patients had CLNM, with an average number of 2.6 ± 3.3 metastatic lymph nodes. A total of 825 (46.5%) patients had LLNM, with an average number of 1.7 ± 2.9 metastatic lymph nodes. Among these patients, 684 (38.6%) had level II/III LNM, and 374 (21.1%) had level IV LNM. “Skip metastasis” was observed in 139 (7.8%) patients (Table 1) (Fig. 1).

**Table 1:** Baseline clinicopathologic characteristics of 1773 PTC patients

	Mean + SD		Mean + SD
Age (years)	42.1 ± 12.4	Number of CLNMs	2.6 ± 3.3
Size (cm)	1.5 ± 1.0	Number of LLNM	1.7 ± 2.9
Sex		CLNMs	
Female	1260 (71.1%)	No	576 (32.5%)
Male	513 (28.9%)	Yes	1197 (67.5%)
Age		LLNM	
< = 55	1289 (84.9%)	No	948 (53.5%)
> 55	230 (15.1%)	Yes	825 (46.5%)
Size (cm)		Level II/III	
≤ 1	862 (48.6%)	No	1089 (61.4%)
1~2	624 (35.2%)	Yes	684 (38.6%)
> 2	287 (16.2%)	Level IV	
Location		No	1399 (78.9%)
Upper	624 (35.2%)	Yes	374 (21.1%)
Non-upper	1149 (64.8%)	pNx	
ETE		pN0	437 (24.6%)
No	1398 (78.8%)	pN1	1336 (75.4%)
Yes	375 (21.2%)		
Multifocality		Hashimoto	
No	1461 (82.4%)	No	1526 (86.1%)
Yes	312 (17.6%)	Yes	247 (13.9%)

*ETE* extrathyroidal extension, *CLNM* central lymph node metastasis, *LLNM* lateral lymph node metastasis, *pN0* pathologic node-negative, *pN1* pathologic node-positive

### Type of lymph node metastasis

**Fig. 2:**
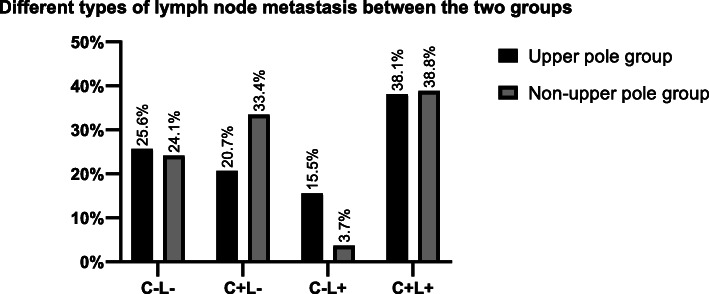
different type of the lymph node metastasis between the two group. C−L−, central lymph node negative with lateral lymph node negative; C + L−, central lymph node positive with lateral lymph node negative; C−L+, central lymph node negative with lateral lymph node positive; C + L+ central lymph node positive with lateral lymph node positive

According to tumour location, the patients were divided into two groups. No metastatic lymph nodes were found in 160 (25.6%) patients and 277 (24.1%) patients in each group. A total of 384 (33.4%) patients in the non-upper pole group experienced metastasis to level VI without lateral neck lymph node involvement (pathologic N1a disease, pN1a), which was more than those in the upper pole group (20.7%). In the upper pole group, 53.68% of patients had LLNM. In contrast, 42.65% of patients in the non-upper pole group had LLNM. Skip metastasis was observed in 97 patients (15.54%) in the upper pole group and 42(3.66%) in the non-upper pole group, with a statistically significant difference (*P* < 0.05) (Table 2 and Fig. 2).

**Table 2:** Lymph node metastasis type in 1773 PTC patients

	Upper pole group	Non-upper pole group	*P* value
*C*−*L*−	160 (25.64%)	277 (24.11%)	<0.001
*C + L*−	129 (20.67%)	384 (33.42%)	
*C*−*L+*	97 (15.54%)	42 (3.66%)	
*C + L+*	238 (38.14%)	446 (38.82%)	

*C*−*L*− central lymph node negative with lateral lymph node negative, *C + L*− central lymph node positive with lateral lymph node negative, *C*−*L+* central lymph node negative with lateral lymph node positive, *C + L+* central lymph node positive with lateral lymph node positive

### Clinicopathological variables associated with LLNM

Clinicopathological differences between LLNM and clinicopathological variables are shown in Table 3. A total of 825 patients (46.5%) had LLNM. Univariate analysis (Table 3) indicated that this condition was significantly associated with sex (*P* < 0.001), tumour size (*P* < 0.001), tumour location (*P* < 0.001), ETE (*P* < 0.001) and CLNM (*P* < 0.001). Multivariate analysis (Table 3) revealed that male sex (adjusted OR (95% CI) 1.486 (1.184–1.867)), tumour location in the upper pole (2.136 (1.707–2.672)), large tumour size (2.496 (1.844–3.380)) and CLNM (4.172 (3.279–5.308)) remained independent significant risk factors (all *P* < 0.05).

**Table 3:** Univariate and multivariate analyses of factors associated with lateral lymph node metastasis in 1773 PTC patients

	Univariate analysis			Multivariate analysis	
	LLNM (−)	LLNM (+)	*P* value	Adjusted OR (95% CI)	*P* value
	*n* = 948	*n* = 825			
Sex			< 0.001^*^		
Female	713 (75.2%)	547 (66.3%)			
Male	235 (24.8%)	278 (33.7%)		1.486 (1.184–1.867)	< 0.001^*^
Age			0.536		
< = 55	673 (85.4%)	616 (84.3%)			
> 55	115 (14.6%)	115 (15.7%)			
Size (cm)			< 0.001^*^		
≤ 1	549 (57.9%)	313 (37.9%)			
1~2	291 (30.7%)	333 (40.4%)		1.707 (1.359–2.144)	< 0.001^*^
> 2	108 (11.4%)	179 (21.7%)		2.496 (1.844–3.380)	< 0.001^*^
Location			< 0.001		
Upper	289 (30.5%)	335 (40.6%)		2.136 (1.707–2.672)	< 0.001^*^
Non-upper	659 (69.5%)	490 (59.4%)			
ETE	160 (16.9%)	215 (26.1%)	< 0.001^*^	1.332 (1.031-1.72)	0.028^*^
Multifocality	152 (16.0%)	160 (19.4%)	0.064		
Hashimoto	136 (14.3%)	111 (13.5%)	0.589		
CLNM	511 (53.9%)	686 (83.2%)	< 0.001^*^	4.172 (3.279–5.308)	< 0.001

*Symbol for *p* < 0.05

*ETE* extrathyroidal extension, *CLNM* central lymph node metastasis, *LLNM* lateral lymph node metastasis, *OR* odds ratio, *CI* confidence interval

### Clinicopathological variables associated with skip metastasis and LLNM

Table 4 shows that the skip metastasis rate was higher in older patients (*P* < 0.001) and in tumours located in the upper pole (*P* < 0.001). In the multivariate analysis, these factors remained independently associated with skip metastasis; the adjusted ORs with 95% CIs were 2.354 (1.522–3.640) and 4.295 (2.885–6.395), respectively.

**Table 4:** Relationships between clinicopathological characteristics and skip metastasis

	Univariate analysis			Multivariate analysis	
	(−)	(+)	*P* value	Adjusted OR (95% CI)	*P* value
Sex			0.411		
Female	1157 (70.8%)	103 (74.1%)			
Male	477 (29.2%)	36 (25.9%)			
Age			< 0.001^*^	2.354 (1.522–3.640)	< 0.001^*^
< = 55	1200 (86.0%)	89 (72.4%)			
> 55	196 (14.0%)	34 (27.6%)			
Size (cm)			0.101		
≤ 1	796 (48.7%)	66 (47.5%)			
1~2	582 (35.6%)	42 (30.2%)			
> 2	256 (15.7%)	31 (22.3%)			
Location			< 0.001^*^	4.295 (2.885–6.395)	< 0.001^*^
Upper	527 (32.3%)	97 (69.8%)			
Non-upper	1107 (67.7%)	42 (30.2%)			
ETE	338 (20.7%)	37 (26.6%)	0.100		
Multifocality	283 (17.3%)	29 (20.9%)	0.292		
Hashimoto	229 (14.0%)	18 (12.9%)	0.728		

*Symbol for *p* < 0.05

*ETE* extrathyroidal extension, *OR* odds ratio, *CI* confidence Interval

## Discussion

Lymph node metastasis is very common in PTC, with an occurrence rate of 30–80% [13, 14]. Even in papillary thyroid microcarcinoma (PTMC), the reported lymph node metastasis rate is up to 50% [9, 15]. In this study, we analysed data from a total of 1773 PTC patients and found different patterns of lymph node metastasis depending on tumour location. PTC was more likely to present a sequential lymph node metastasis pattern (first to the central and then to the lateral compartments) when tumours were located in the middle or lower pole, which is in agreement with two previous studies [16, 17]. The rate of skip metastasis was also significantly low. However, when tumours were located in the upper pole, they tended to metastasize to the lateral lymph nodes more frequently. This finding indicates that upper pole tumours more likely involve the lateral compartment than tumours located at other positions, which has also been reported in several studies [18, 19]. Another study [20] has shown that upper pole tumours seem to be at a high risk for malignancy. Jasim et al. [21] argued that upper thyroid nodules had a higher risk for malignancy, with an OR of 1.8 (1.2–2.7), followed by middle and lower thyroid nodules in a review of 3313 adult patients. However, there were no clear recommendations on whether lateral LND should be performed when the tumour is located in the upper pole [5, 22]. If tumour location was not considered, only central compartment lymphadenectomy was performed, and the potential risk of lateral lymph metastasis might have been ignored. In addition to the previously confirmed influencing factors, tumour location should be evaluated seriously. LND should be managed appropriately during surgery, even in small tumours located in the upper pole, to avoid local recurrence and distant metastasis.

Tumours located in the upper pole were identified as an independent risk factor for skip metastasis in our study. Luo et al. [23] reviewed 1031 PTMC patients and established that tumour location in the upper pole was closely correlated with lateral cervical lymph node metastasis and not CLNM. These unique phenomena are conventionally called “skip metastasis” in most studies. However, given the current retrospective study and anatomical support of lymphatic spread, this nomenclature may not be appropriate. There might be an independent lymphatic drainage pathway in the upper pole of the thyroid tumour that directly drains to the lateral region and into the deep vein without passing through the central region. In other words, the lateral lymph node might be the first lymph drainage station for an upper pole tumour. Likhterov et al. [16] also supported the exclusive lymphatic pathway of the upper pole using lymphatic anatomic studies. Jianyong et al. [12] used carbon nanoparticles injected into the upper pole of the thyroid lobe, and 70% of tumours showed skip metastasis (black staining), in which all of the patients showed level II skip metastasis. This potential lymphatic drainage pathway may cause occult lymph node metastasis and should be carefully considered during surgical intervention. This hypothesis needs confirmation through more prospective research and anatomical evidence.

Our study also demonstrated that male sex, a large tumour size, ETE and CLNM were significantly correlated with LLNM by multivariate analysis. A random-effects model with 16 studies [10] showed that male patients had a poorer OR (1.72) than female patients with LLNM, which was in line with recent research [24]. Many previous studies have indicated that a large tumour size is associated with LLNM and leads to later tumour staging [24, 25]. Kim et al. [26] reported that a tumour size > 0.5 cm was an independent predictor of LLNM (adjusted OR 1.295) according to a review of 5656 PTMC patients, which is consistent with our study. In the present study, ETE was associated with LLNM. Kim et al. [27] found that ETE was an admittedly important prognostic factor for LLNM. However, in patients with skip metastasis, only age and tumour location remained statistically significant, which was congruent with our previous study [28]. A recent meta-analysis of 13 articles [29] also found that older age and upper pole tumours were risk factors for LLNM.

The most common metastatic site of PTC is the central compartment, which has been reported to be an independent contributor to LLNM [25, 30]. This conclusion confirms the main lymph node drainage pathway of the thyroid. CLNM, which was the main contributor among all patients in our study, was observed at a lower rate in the upper pole group compared with the non-upper pole group. This result further indicates that there might be a direct lymph pathway from the upper pole of the thyroid to the lateral compartments, consistent with a previous report [31].

There is no official standard or acknowledged anatomical mark for the location of thyroid tumours. In our study, tumour location was determined with ultrasound and findings during the operation. There should be a concise standard based on imaging (US, CT, etc.) and pathology in future recommendations.

There were several potential limitations to this study. Several clinicopathological features, such as histological subtype, Braf mutation and lymphovascular invasion, were not provided. Furthermore, we are examining more detailed pathological reports and long-term follow-up data.

## Conclusion

The lymphatic drainage pathway of PTC is complex. The extent of LND in each patient should be individualized. When the tumour is located in the upper pole of the thyroid, it is more likely to have LLNM than when a tumour is not located in the upper pole. The risks and benefits of preventive lateral LND should be discussed with the patient.

## Data Availability

The datasets used and/or analysed during the current study are available from the corresponding author on reasonable request.

## Abbreviations

ETE: Extrathyroidal extension
CLNM: Central lymph node metastasis
LLNM: Lateral lymph node metastasis
pN0: Pathologic node-negative
pN1: Pathologic node-positive
PTC: Papillary thyroid carcinoma
ORs: Odds ratios
CIs: Confidence intervals
TT: Total thyroidectomy
FNAB: Fine-needle aspiration biopsy
HT: Hashimoto’s thyroiditis
